# Infected Tibial Plateau Open Reduction Internal Fixation Treated Using External Fixation and a Gastrocnemius Flap: A Case Report

**DOI:** 10.7759/cureus.46750

**Published:** 2023-10-09

**Authors:** Nicolae Angan, Boris Feghiu, Ioana Dumitrescu, Valentin Feghiu

**Affiliations:** 1 Trauma and Orthopaedic Surgery, Mater Misericordiae University Hospital, Dublin, IRL; 2 Trauma and Orthopaedic Surgery, Elias Emergency University Hospital, Bucharest, ROU; 3 Plastic Surgery, Elias Emergency University Hospital, Bucharest, ROU; 4 Trauma and Orthopaedic Surgery, Territorial Medical Association, Chişinău, MDA

**Keywords:** biofilm, complications, infected non-union, implant-related infection, infection after fracture fixation

## Abstract

Here, we describe the case of an 80-year-old female patient with type II insulin-dependent diabetes mellitus with a left proximal tibia fracture. Open reduction internal fixation was performed using a locking plate. After the surgical site infection, the plate was removed and negative-pressure wound therapy was applied. The bone was covered with a vastus medialis muscle flap, and a split-thickness skin graft and external fixation using an Ilizarov device was performed as the definitive treatment.

## Introduction

Tibial plateau fractures are complex injuries that often require surgical intervention to restore anatomy and facilitate optimal healing [1]. However, the presence of complications, such as surgical site infections (SSIs), can significantly impede the success of the initial treatment. In this case report, we present the management of an 80-year-old female patient with type II insulin-dependent diabetes mellitus (IDDM) who developed an SSI following open reduction internal fixation (ORIF) of a left proximal tibia fracture [2]. The subsequent treatment involved the removal of the hardware, debridement, negative-pressure wound therapy (NPWT), and the implementation of an external fixation device along with a gastrocnemius flap and skin graft to cover the exposed bone.

Diabetes mellitus, particularly IDDM, is a well-known risk factor for impaired wound healing and increased susceptibility to infections. In the case presented, the patient’s pre-existing IDDM posed a significant challenge in managing the infection and promoting bone healing.

This case report aims to highlight the challenges faced in managing infected tibial plateau fractures in patients with underlying comorbidities, such as IDDM. It underscores the importance of a multidisciplinary approach, encompassing orthopedic expertise, plastic surgery techniques for bone coverage, and meticulous wound management. By sharing this case, we hope to contribute to the existing body of knowledge, providing insights into the successful treatment of complex fractures complicated by infection and underlying systemic conditions.

Although this report represents a single case, it offers valuable insights into the therapeutic strategies employed and the subsequent clinical outcomes observed. The subsequent sections will provide a detailed account of the patient’s clinical history, the surgical procedures performed, and the postoperative management strategies adopted. The aim is to contribute to the understanding and treatment of infected tibial plateau fractures, particularly in patients with significant comorbidities that impact the healing process.

## Case presentation

An 80-year-old Caucasian woman was brought to the emergency department complaining of left knee pain. The patient lived alone and after falling tried to bear weight on the left leg and called emergency services four days after the injury. Previously, she was independently ambulating without walking aids, but after the fall she was unable to bear weight on the left leg. The patient was suffering from type II IDDM, sight disorder, and chronic renal disease.

**Figure 1:**
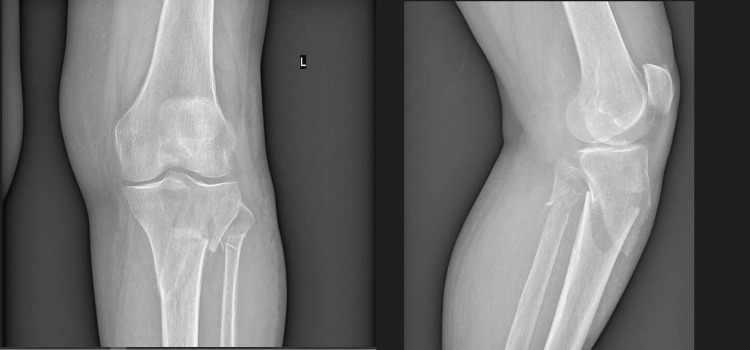
X-ray of the left proximal tibia showing anteroposterior and lateral views.

On physical examination significant knee effusion was present and the patient was not able to bend the knee. The plain X-rays showed a proximal tibia fracture (Figure 1).

**Figure 2:**
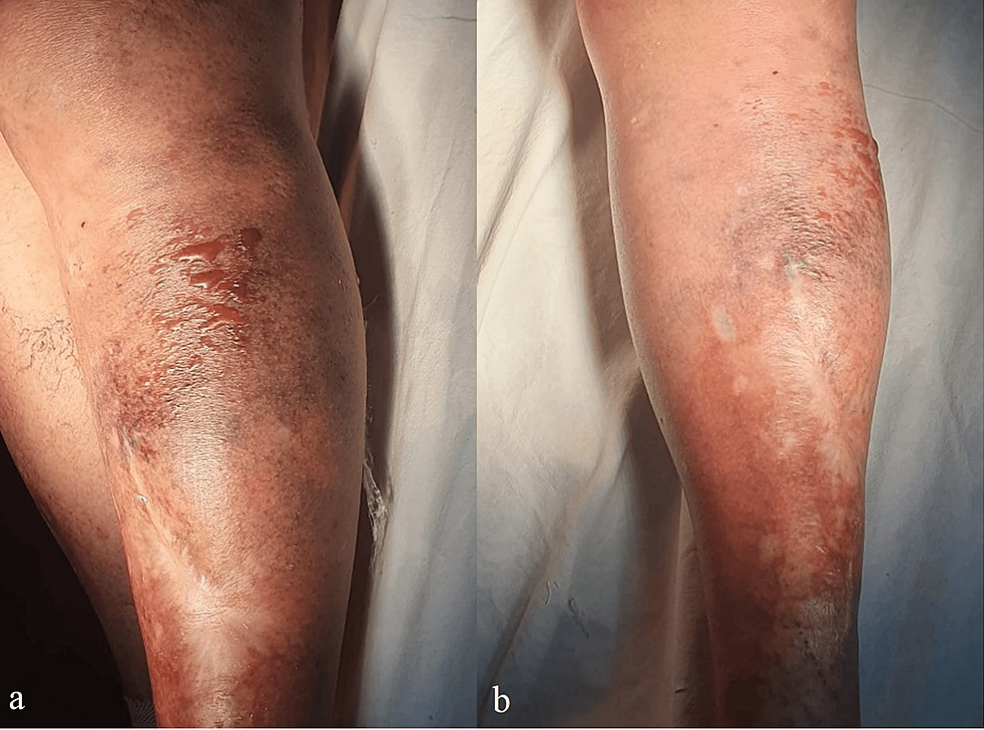
Lateral (a) and anterior (b) view of the left leg (proximal third).

A long leg backslab was applied and the patient was admitted for surgical treatment. The following day the skin was inspected and serous blisters on the lateral side of the left leg (proximal third) were noted (Figure 2).

**Figure 3:**
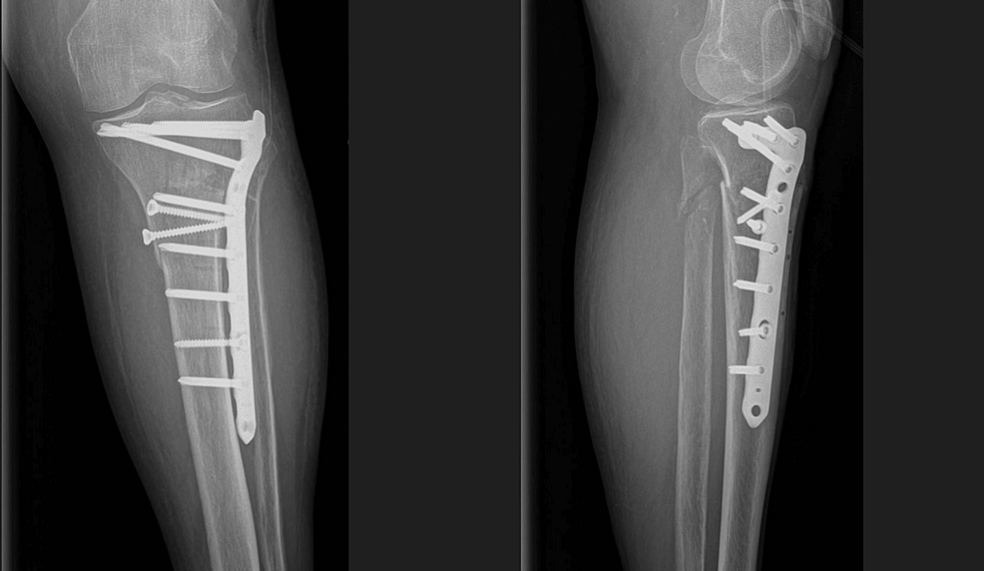
X-ray of the left proximal tibia showing anteroposterior and lateral views.

Proximal tibia ORIF using an L-shaped locking plate was performed the day after admission. The anterolateral approach was used to avoid the serous blisters. No tourniquet was applied during the surgery. Soft tissues were swollen, and it was difficult to close the wound after plate fixation. Plain radiographs were obtained after the surgery (Figure 3).

**Figure 4:**
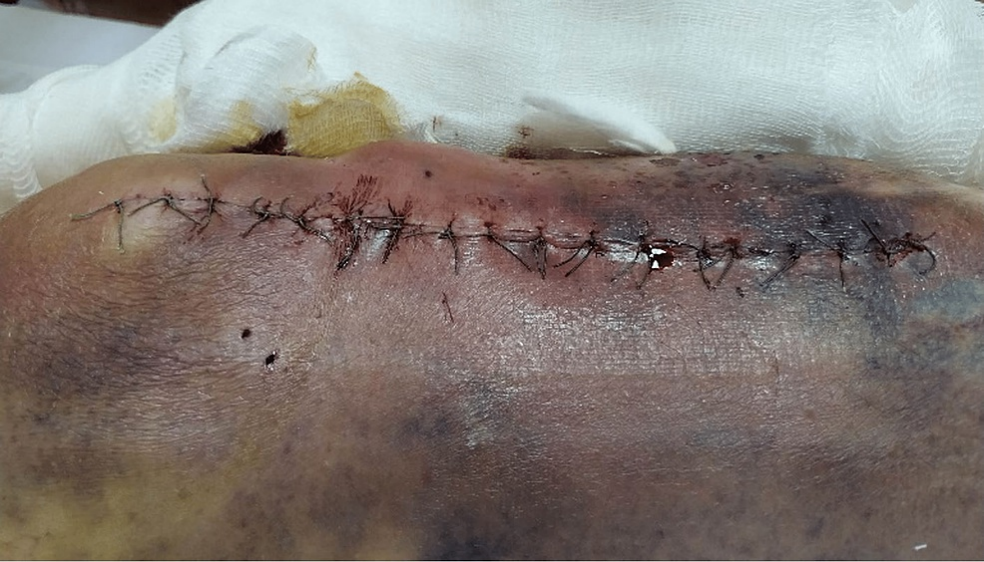
Wound inspection 24 hours after left proximal tibia open reduction internal fixation.

**Figure 5:**
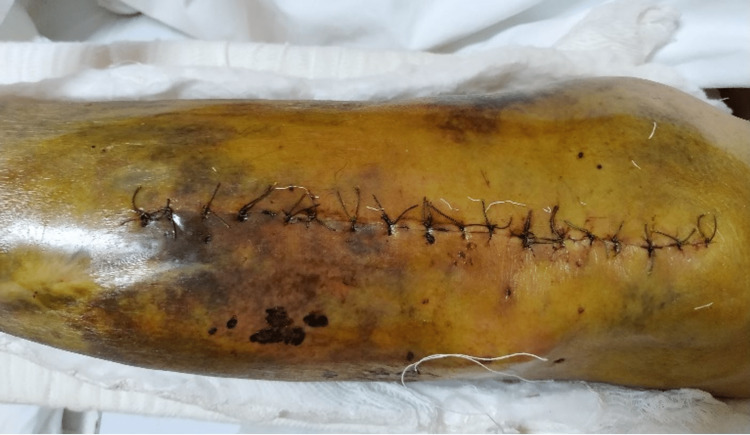
Wound inspection 10 days after left proximal tibia open reduction internal fixation.

The wound was satisfactory 24 hours and 10 days after the surgery (Figures 4, 5). 

**Figure 6:**
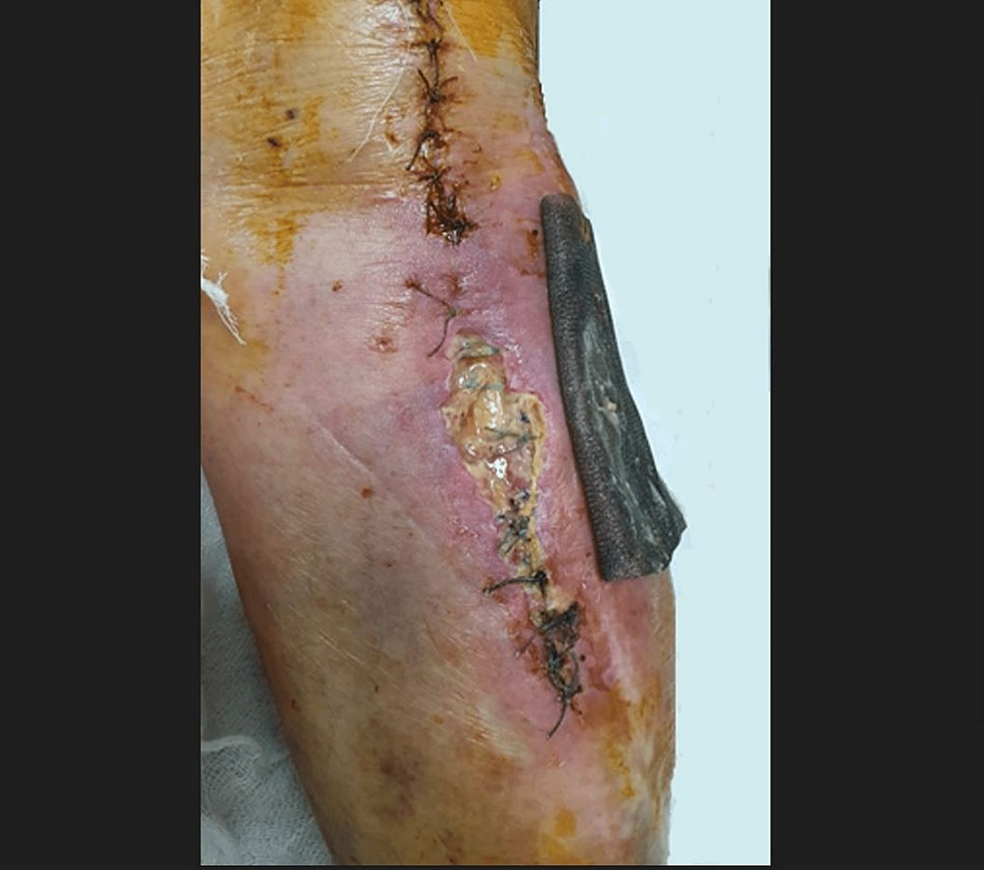
Four weeks after left proximal tibia open reduction internal fixation showing purulent discharge and wound dehiscence.

Three weeks after plate fixation the patient developed redness, swelling, and wound dehiscence. Vital signs were stable on admission. There was approximately 7 cm of dehiscence in the middle part of the wound and purulent discharge (Figure 6).

**Figure 7:**
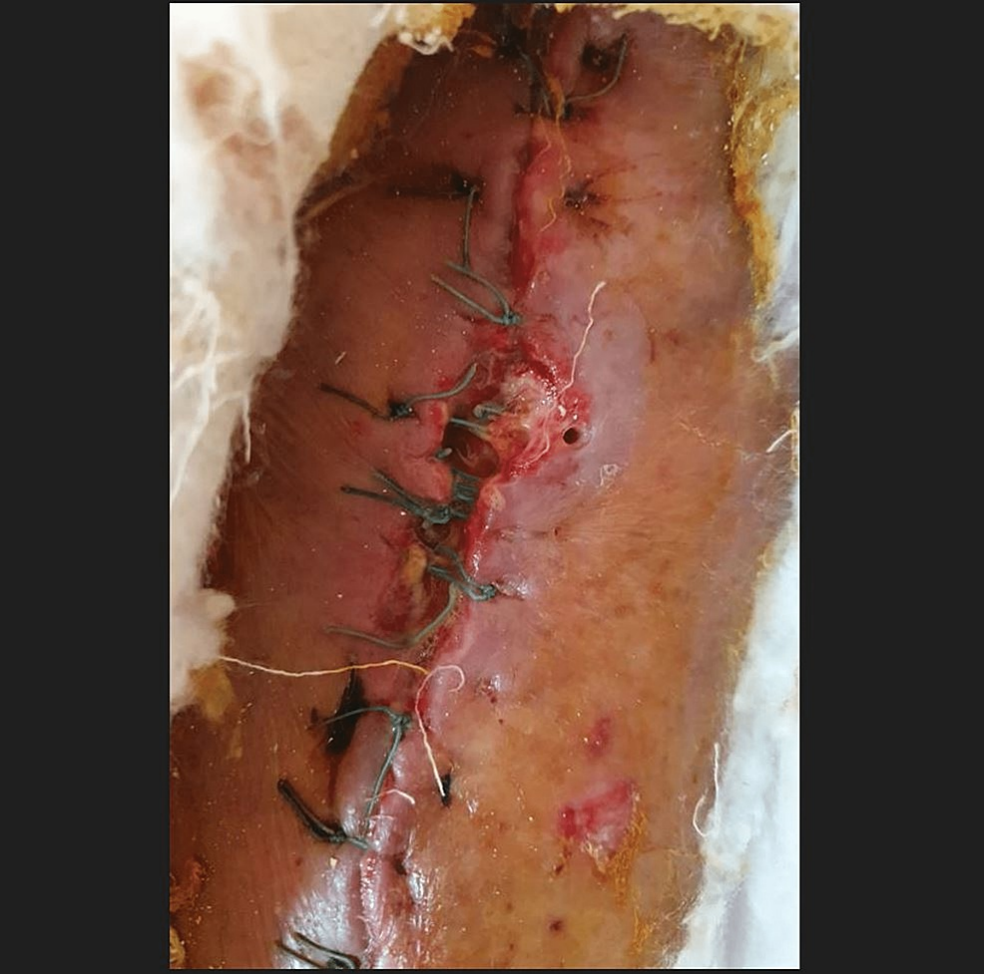
Two days after left proximal tibia removal of hardware.

The patient underwent removal of metal, followed by extensive excisional debridement involving the removal of devitalized skin, muscle, and bone. Soft tissue was taken for culture and sensitivity and broad-spectrum antibiotherapy was started. Subsequently, the wound was sutured. Two days later, the presence of wound dehiscence was observed (Figure 7).

**Figure 8:**
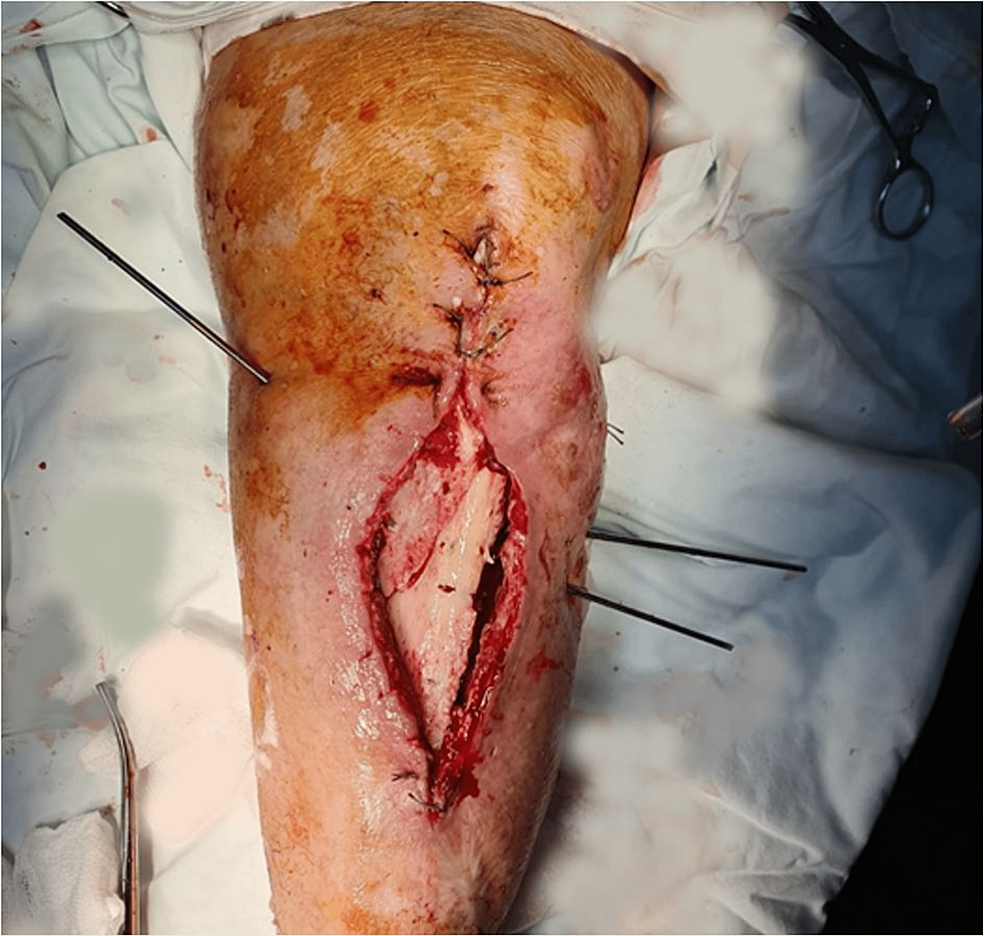
Debridement of necrotic tissue and K-wire fixation (intraoperative image).

Laboratory results including erythrocyte sedimentation rate and C-reactive protein were elevated at 120 mm/hour and 160 mg/L, respectively. Aerobic and anaerobic cultures from wound drainage identified *Staphylococcus aureus* with sensitivity for oxacillin, gentamicin, erythromycin, clindamycin, minocycline, rifampicin, vancomycin, linezolid, and teicoplanin. The patient was started on intravenous antibiotic therapy with oxacillin 1 g every six hours for seven days without decreasing inflammatory markers. Extensive excisional debridement was performed. Additionally, K-wire fixation and NPWT were applied (Figure 8).

A second culture was taken from wound drainage and Enterococcus faecalis was identified. The patient received intravenous antibiotic therapy with amoxicillin and clavulanic acid 1.2 g every eight hours for seven days, followed by a decrease in erythrocyte sedimentation rate and C-reactive protein at 102 mm/hour and 91 mg/L, respectively. There was no fever spike and no blood cultures were taken.

**Figure 9:**
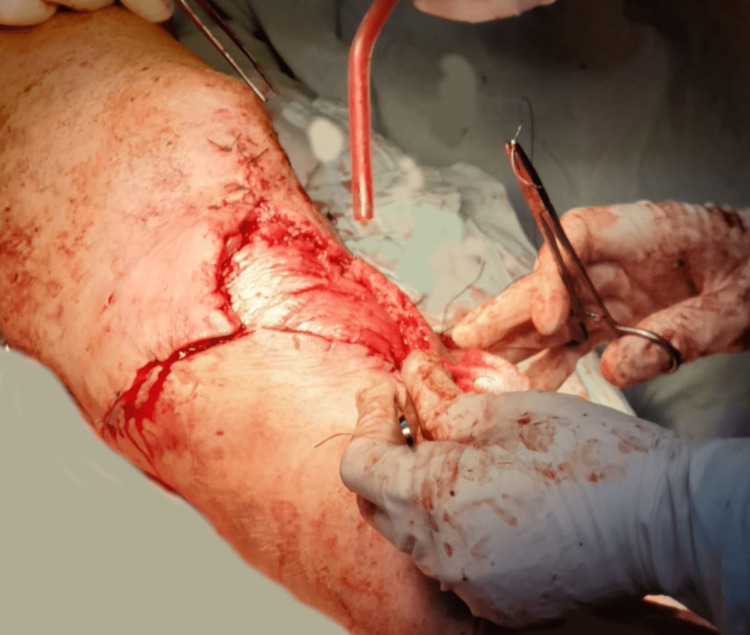
Medial gastrocnemius muscle flap and split-thickness skin graft.

After five days, NPWT was removed and the bone was covered with a medial gastrocnemius muscle flap. The skin graft was harvested from same-side tight and split-thickness skin graft was performed (Figure 9).

**Figure 10:**
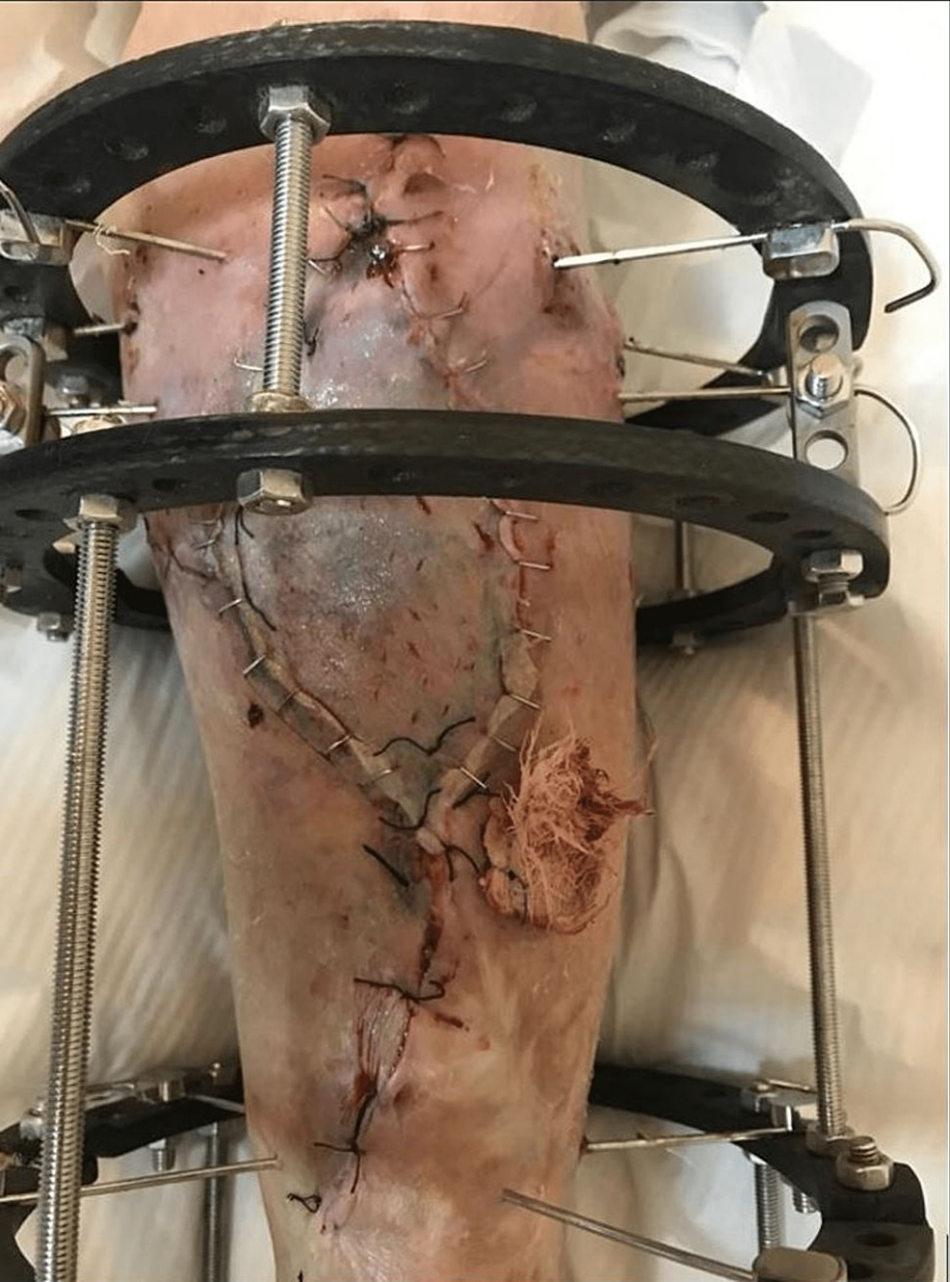
External fixation of the left proximal tibia using an Ilizarov device.

**Figure 11:**
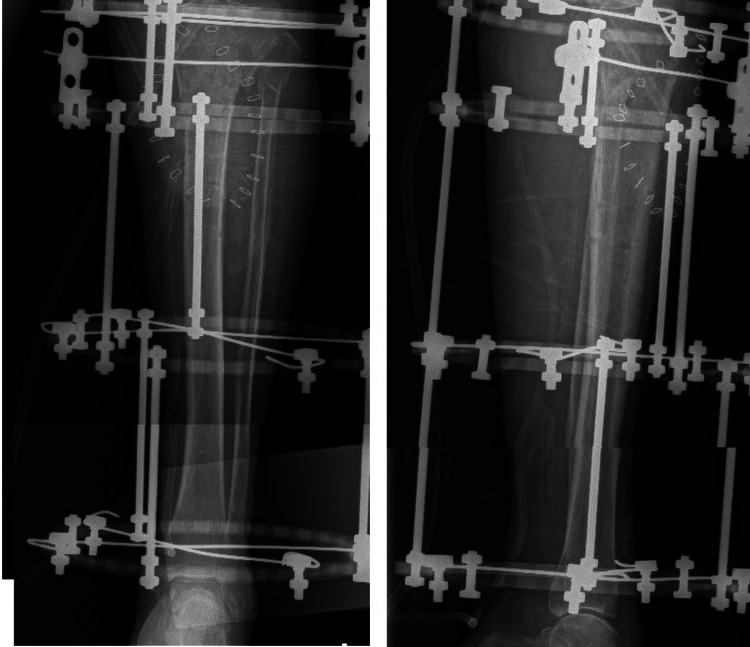
Plain radiographs showing anteroposterior and lateral views after external fixation.

The fracture was fixed with an Ilizarov device and the patient was mobilized with full weight-bearing the following day (Figure 10). Figure 11 shows the anteroposterior and lateral views of the Ilizarov device.

**Figure 12:**
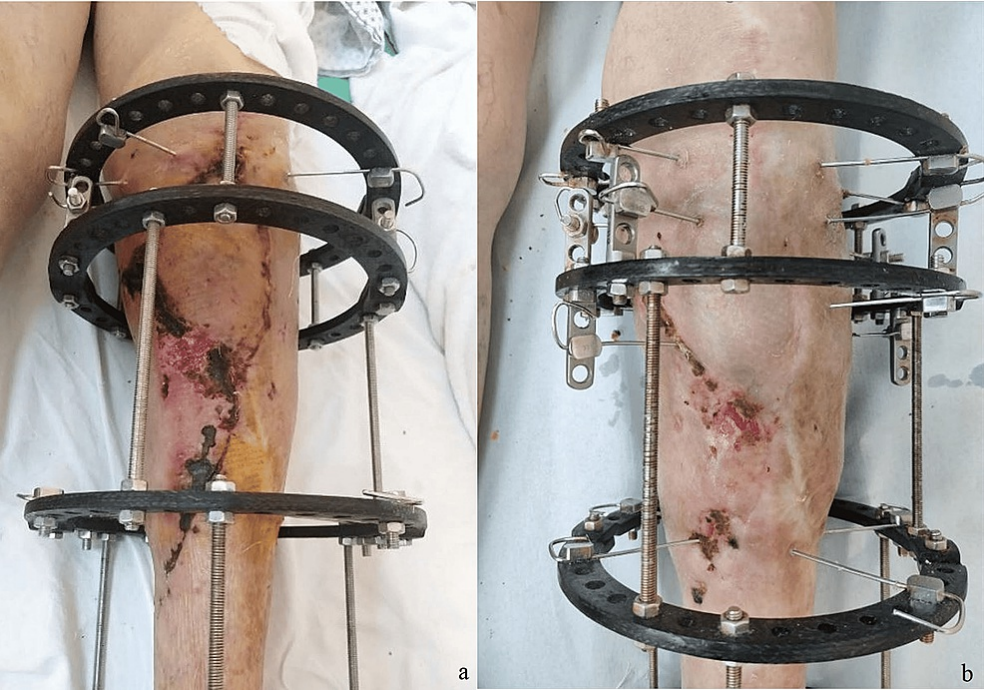
Four (a) and eight (b) weeks after medial gastrocnemius flap and split-thickness skin graft.

The split-skin graft had integrated well on anterior leg wound, as can be seen in photos taken at four weeks (Figure 12a) and eight weeks (Figure 12b) after skin grafting.

**Figure 13:**
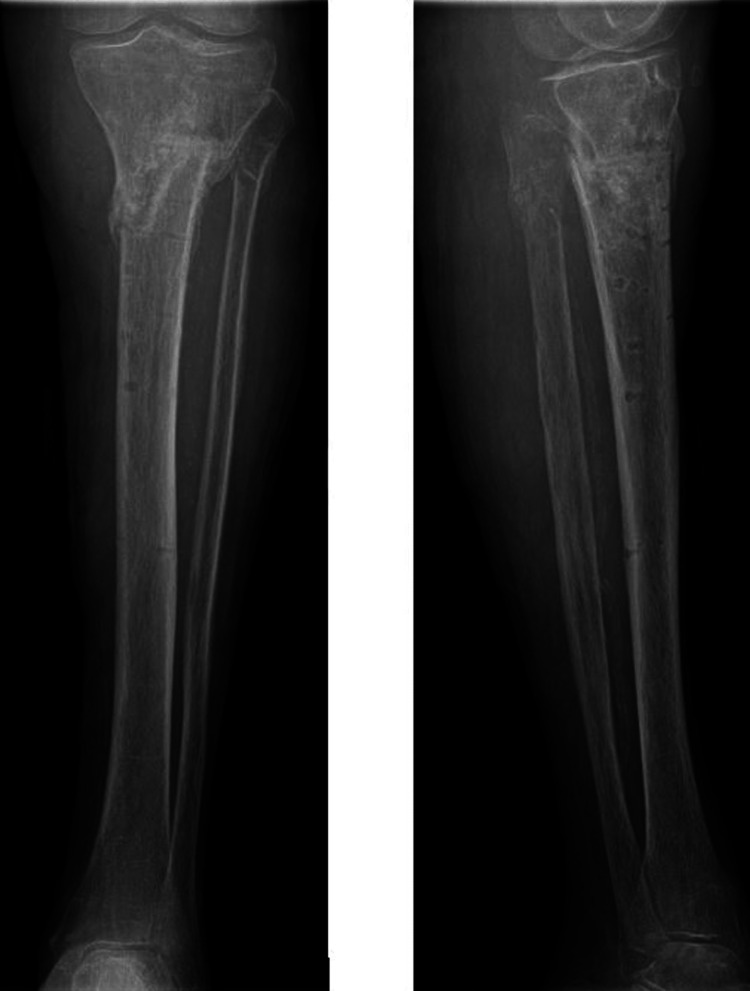
Anteroposterior and lateral X-rays of the left tibia.

After five months, the Ilizarov device was removed. Anteroposterior and lateral radiographs were taken (Figure 13). Partial weight-bearing was allowed.

## Discussion

We present the case of a patient with Schatzker VI [1,2] tibial plateau fracture treated with a locking plate. The present case was complicated with additional soft tissue injury due to late presentation to the emergency department (four days after the injury) and weight bearing on the affected limb.

ORIF is a gold standard treatment for tibial plateau fractures [3]. It allows anatomical reduction of the articular surface. Complex articular fractures can be treated by ring external fixators and minimally invasive osteosynthesis (EFMO) or ORIF [4]. EFMO has been advocated as a way to minimize ORIF complications and can be related to suboptimal articular reduction; however, outcomes analysis shows results that are equal to or even superior to ORIF [5,6].

It is safe to place incisions around intact blisters. It is also safe for the incision to go through a clear-filled blister, while it is advised to avoid blood-filled blisters due to their greater morbidity [1-3].

Infection after fracture fixation (IAFF) is one of the most feared and challenging complications in the treatment of musculoskeletal trauma patients, sometimes requiring several revisions and often with a worse outcome [7,8]. Despite the prophylaxis and treatment of IAFF, the persistence of the problem suggests that they are not effective, and further improvements should be sought.

Surgical and medical treatment concepts currently applied to IAFF have been adopted from prosthetic joint infections (PJIs). In contrast to PJIs [9], there are no available standard criteria and a lack of consensus regarding the definition of IAFF. There is a widely accepted classification scheme of IAFF [10,11], even though there is a lack of a clear definition.

Roth classified IAFF in the 1980s according to the time of onset into the following three groups: those with an early (less than two weeks), delayed (2-10 weeks), and late-onset (more than 10 weeks) infection [11,12]. This classification is important because it affects treatment decisions made by physicians and has been widely adopted [10].

In this case, the patient developed an infection within the 2-10-week interval which classifies it as delayed. As a risk factor for the infection, the patient had type II diabetes [13]. *Staphylococcus aureus* is the most commonly identified bacteria in bone infections [14]. The question is whether the *Staphylococcus aureus* isolated from wound drainage is a contaminant. We do think it is because of the lack of efficiency in sensitive antibiotic therapy.

## Conclusions

This case report serves as a reminder of the complexities associated with managing infected tibial plateau fractures, especially in patients with underlying comorbidities. In isolated cases, external fixation and delayed ORIF can be considered. The utilization of NPWT, gastrocnemius muscle flap, and external fixation demonstrates a promising treatment strategy for achieving successful outcomes in such challenging cases. Continued research and experience sharing in this field will further enhance our understanding and improve the management of infected fractures, ultimately benefiting patients with similar presentations.
